# Comprehensive Assessment of Maize Aflatoxin Levels in Eastern Kenya, 2005–2007

**DOI:** 10.1289/ehp.1003044

**Published:** 2011-08-15

**Authors:** Johnni H. Daniel, Lauren W. Lewis, Yanique A. Redwood, Stephanie Kieszak, Robert F. Breiman, W. Dana Flanders, Carlos Bell, John Mwihia, George Ogana, Sopiato Likimani, Masja Straetemans, Michael A. McGeehin

**Affiliations:** 1 Centers for Disease Control and Prevention, Atlanta, Georgia, USA; 2 Centers for Disease Control and Prevention, Kenya, Nairobi, Kenya; 3 Food and Drug Administration, Rockville, Maryland, USA; 4 Kenya National Public Health Laboratory, Nairobi, Kenya; 5 Kenya Ministry of Public Health and Sanitation, Nairobi, Kenya; 6 KNCV Tuberculosis Foundation, The Hague, the Netherlands

**Keywords:** aflatoxicosis, aflatoxin, contamination, Kenya, maize

## Abstract

Background: Aflatoxin, a potent fungal toxin, contaminates 25% of crops worldwide. Since 2004, 477 aflatoxin poisonings associated with eating contaminated maize have been documented in Eastern Kenya, with a case-fatality rate of 40%.

Objective: We characterized maize aflatoxin contamination during the high-risk season (April–June) after the major harvests in 2005, 2006 (aflatoxicosis outbreak years), and 2007 (a non-outbreak year).

Methods: Households were randomly selected each year from the region in Kenya where outbreaks have consistently occurred. At each household, we obtained at least one maize sample (*n* = 716) for aflatoxin analysis using immunoaffinity methods and administered a questionnaire to determine the source (i.e., homegrown, purchased, or relief) and amount of maize in the household.

Results: During the years of outbreaks in 2005 and 2006, 41% and 51% of maize samples, respectively, had aflatoxin levels above the Kenyan regulatory limit of 20 ppb in grains that were for human consumption. In 2007 (non-outbreak year), 16% of samples were above the 20-ppb limit. In addition, geometric mean (GM) aflatoxin levels were significantly higher in 2005 (GM = 12.92, maximum = 48,000 ppb) and 2006 (GM = 26.03, maximum = 24,400 ppb) compared with 2007 (GM = 1.95, maximum = 2,500 ppb) (*p*-value < 0.001). In all 3 years combined, maize aflatoxin levels were significantly higher in homegrown maize (GM = 17.96) when compared with purchased maize (GM = 3.64) or relief maize (GM = 0.73) (*p*-value < 0.0001).

Conclusions: Aflatoxin contamination is extreme within this region, and homegrown maize is the primary source of contamination. Prevention measures should focus on reducing homegrown maize contamination at the household level to avert future outbreaks.

Aflatoxins are a group of mycotoxins produced mainly by *Aspergillus* spp. fungi that grow on various crops such as maize, nuts, and other grains (Patten 1981). Chronic dietary exposure to low doses of aflatoxins is a known risk factor for liver cancer and may also affect protein metabolism and immunity, thus worsening infectious diseases and malnutrition (Williams et al. 2004). Ingesting highly contaminated products results in severe, acute hepatitis known as aflatoxicosis; symptoms include vomiting, jaundice, and abdominal pain and can lead to fulminant liver failure and death. No specific treatment has been found for acute aflatoxicosis. With supportive care, the case fatality rate for acute poisoning ranges from 25 to 40% (Azziz-Baumgartner et al. 2005; Eaton and Groopman 1993).

Multiple risk factors increase human exposure to dietary aflatoxins. Inadequate drying and storage under damp conditions are primary factors that lead to aflatoxin production and grain contamination (Wilson and Payne 1994). In addition, drought conditions and insect invasion can weaken crops and make them susceptible to contamination (Sinha and Sinha 1992).

Subsistence farmers in developing countries are perhaps the most at risk. They often lack the capacity to protect crops against aflatoxin contamination. In addition, food insecurity due to drought and other causes of crop failure seem to contribute to behaviors that increase risk of exposure. Finally, 60–85% of the population in the developing world are subsistence farmers and are not protected by commercial food safety regulation (Wild 2007). As a result, there are considerable disparities in aflatoxin exposure between people living in developed and developing countries.

Perhaps the most extreme exposure to aflatoxin documented globally occurs in the southern region of Kenya’s Eastern province [Azziz-Baumgartner et al. 2005; Centers for Disease Control and Prevention (CDC) 2004; Lewis et al. 2005; Ngindu et al. 1982]. Outbreaks of acute aflatoxicosis recur in the region, specifically, in two adjacent districts, Makueni and Kitui, that have consistently been the epicenter of aflatoxicosis outbreaks in the region (Azziz-Baumgartner et al. 2005; Ngindu et al. 1982). Makueni and Kitui are located approximately 130 km southeast of Nairobi and have a combined estimated population of 1,591,343 (Kenya Integrated Household Budget Survey 2005/2006). The districts lie in a drought-prone region that consists of small-scale subsistence farms and where maize is the main crop produced and consumed (Lewis et al. 2005).

The first recorded outbreak in this area was in 1981, resulting in 20 cases and 12 deaths. Consumption of contaminated maize was established as the source of aflatoxin exposure. The maize grains from households where severe and fatal illness had occurred contained aflatoxin levels up to 12,000 ppb (Ngindu et al. 1982). In April 2004, the most severe aflatoxicosis outbreak ever reported occurred in this region. That outbreak resulted in 317 cases and 125 deaths and was due to contamination of improperly stored maize (Azziz-Baumgartner et al. 2005; CDC 2004).

Epidemiologic investigations of these previous outbreaks identified consistent characteristics of aflatoxicosis outbreaks in the region. Maize was the established source of aflatoxin exposure, and the season between April and June (4–8 weeks after the major crop has been harvested and stored) was established as the high-risk season when acute aflatoxicosis outbreaks consistently occurred (Azziz-Baumgartner et al. 2005; Mwihia et al. 2008; Ngindu et al. 1982; CDC 2004). Historically, outbreaks have highlighted the extreme aflatoxin exposure in this region. However, the extent of aflatoxin contamination is unknown, and community awareness of aflatoxin and aflatoxicosis and other factors that may influence exposure in the affected geographic locations have not been well characterized.

After the large outbreak in 2004, the CDC (2004) and the government of Kenya initiated a public health response that included hospital-based, clinical disease surveillance for acute aflatoxicosis in the four hospitals serving the high-risk districts (Makueni and Kitui). Cases were reported to public health officials by clinical care providers along with other reportable diseases. Because there is no diagnostic test for aflatoxicosis, cases were defined as acute hepatic inflammation of unknown etiology (where viral and other causes have been ruled out). In addition, the Kenyan government, along with the Food and Agriculture Organization (FAO), initiated a public education campaign on aflatoxin prevention. Finally, household maize surveys during the high-risk season (April–June) were initiated in Makueni and Kitui to understand the distribution of aflatoxin in maize and to guide urgent interventions to control aflatoxin exposure and prevent aflatoxicosis.

This report presents an analysis of household maize survey data collected in 2005, 2006, and 2007 as part of the urgent public health responses. The data were originally collected to guide and target maize replacement efforts and other interventions. The objectives of the present analysis were to describe the distribution and extent of maize aflatoxin contamination and to identify associated factors, such as the source of the contaminated maize (i.e., maize homegrown on the farm where consumed, maize purchased from a store or market, or maize received as part of relief efforts), amount of maize produced, and aflatoxin awareness, among subsistence farmers living within the Makueni and Kitui districts of the Eastern Province of Kenya.

## Methods

*Study design.* We conducted an analysis of data collected during the household maize surveys in 2005, 2006, and 2007. We employed a cross-sectional study design in all 3 years. The data set includes interview data collected at each household along with one or more household maize samples for aflatoxin analysis. The surveys were conducted in Makueni and Kitui districts between April and June (high-risk season). Aflatoxicosis outbreaks were occurring in the region during the 2005 and 2006 surveys, but not during the 2007 survey. The 2005 and 2006 surveys included households systematically selected because one or more cases of aflatoxicosis resided in the household. These aflatoxicosis case households were identified through the ongoing hospital-based, clinical disease surveillance described above.This study was approved by the Centers for Disease Control and Prevention Institutional Review Board.

*Sample selection.* In 2005, 2006, and 2007, we conducted the maize household surveys in the divisions (geographic government units within districts) within Makueni and Kitui districts where cases of aflatoxicosis had occurred during the current outbreak or previous outbreaks. Each year villages were randomly selected from these divisions. However, the methods used to obtain a random sample of villages and the number of villages selected varied slightly from year to year. These variations reflect differences from year to year in capacity, available resources, and public health response needs.

In 2005, we selected a total of 36 villages. We selected half (18) from a list of villages where a person with aflatoxicosis resided, as reported through hospital-based disease surveillance and obtained from the local public health officers. The other half (18) of the villages were randomly selected from the affected region but where no cases of aflatoxicosis were reported. Specifically, using census data, we randomly selected 18 sublocations (the smallest geographic government unit given in Kenya census data) from a list of sublocations within the affected divisions. One village was selected from each sublocation. In 2006, we selected a total of 18 villages from 18 randomly selected sublocations within affected divisions without regard to whether an aflatoxicosis case resided in the village. In 2007, we selected a total of 24 villages without regard to whether a case resided in the village. The methods used to obtain a random sample differed in 2007 from 2005 and 2006. In 2007, we randomly selected points using a geographical information system (ArcMap^®^ 9.0; ESRI, Redlands, CA, USA) random point generator within the affected divisions and selected a village based on proximity to a randomly selected point.

In 2005, 2006, and 2007, we used the same method to select households within the selected villages. If a case resided in the village, the case household was included in the survey. To select the other (noncase) households in the village, we randomly chose a direction from the village center by spinning a pencil and walking in the direction of the pencil point, a method borrowed from the World Health Organization Expanded Program on Immunization (Lemeshow and Robinson 1985). In each year, we enrolled the maximum number of households that were logistically feasible to visit each day.

*Questionnaire.* In all 3 years, we administered a standardized questionnaire to the head of household (18 years of age or older) after obtaining informed consent. We asked questions pertaining to the source of maize currently being eaten by members in the household. We defined the sources of maize as *a*) homegrown: maize grown on the household farm where it will be consumed; *b*) purchased: maize bought from local market vendors, which could be locally grown, imported, or a blend of both; and *c*) relief: maize provided by the Kenyan government or a nongovernmental organization as part of relief efforts. We also asked about maize crop production and assessed the amount of homegrown maize produced from the current harvest by asking household members to report the number of kilograms or number of 90-kg bags of maize produced during the current harvest and to report alternative food sources. We asked about awareness of aflatoxin contamination and aflatoxicosis through multiple questions about drying and storing practices and knowledge of the potential causes of jaundice. In addition, we asked about the health of family members, including questions about any current symptoms of jaundice among household members and other symptoms that could be attributed to aflatoxicosis.

*Maize collection.* We attempted to collect at least one sample of maize from each selected household for analysis. In all 3 years, we sampled the maize being consumed by household members at the time of the study, regardless of source. Maize samples were classified as homegrown, purchased, or relief maize. If a household had maize from more than one source, we requested a sample from each one. Most households store maize in 90-kg bags. We used a process recommended by the FAO for maize collection that has not been statistically verified (Njapau 2008). Specifically, we took multiple samples from different parts of one bag and combined them to produce a representative 1-kg sample for analysis. If a household had several bags from the same source, we collected grains from a random sample of bags to create the 1-kg sample. To reduce the risk of cross-contamination between households, we collected maize samples using tools that were present at the household. We stored and transported samples for analysis in paper bags to control moisture content. We provided each household with a 2-kg bag of maize flour for their willingness to provide a sample.

*Maize aflatoxin analysis.* We analyzed maize samples for aflatoxin levels at the National Public Health Laboratory in Nairobi, Kenya, using the immunoaffinity column (AflaTEST; VICAM, Milford, MA, USA) method 977.16 (Association of Official Analytical Chemists 2005) that was provided by VICAM. We mixed ground maize (50 g) that passed through a no. 20 sieve with 100 mL methanol:water mixture (80:20) and 5 g sodium chloride before filtration. We diluted 10 mL of the extract with 40 mL distilled water that was filtered again through a microfiber glass filter into a glass syringe. We then passed 2 mL of the twice-filtered mixture through the immunoaffinity column at a rate of 1–2 drops/sec. We washed the column with water and recovered the aflatoxins using 1 mL methanol. We read the methanol extract using a calibrated VICAM Series-4 fluorometer (VICAM) set at 360 nm excitation and 450 nm emissions. This method has an aflatoxin recovery of ≥ 85% and a detection limit of 1 ppb.

*Statistical analysis of the data.* We calculated descriptive statistics, including the number and percentage of maize samples > 20 ppb, the Kenyan regulatory limit for aflatoxins in grains for human consumption (FDA 2000), and geometric mean (GM) aflatoxin levels. When estimating GMs, the values that were less than the limit of detection (LOD) were substituted with the LOD divided by the square root of two. The *p*-value used was 0.05. We used mixed linear models to investigate the association between aflatoxin concentration in maize samples and selected items from the questionnaire, including source of the maize, geographic location of the household (e.g., division and village), and questions measuring aflatoxin awareness. In these analyses, aflatoxin concentration levels were log-transformed to normalize. Because the village selection methods varied slightly between years, we conducted analyses separately by year. A random effect of village within division group was included in each model. In addition, we combined the data from 2005, 2006, and 2007 to calculate summary estimates of aflatoxin contamination, source, and awareness data adjusted for year. We calculated least square means for the fixed effects specified in the models (e.g., year and source). We analyzed aflatoxin concentration and questionnaire data using SAS software (version 9.2; SAS Institute, Cary, NC, USA).

## Results

*Description of study sample.* In the 3 years combined, we surveyed a total of 99 villages: 46 in the Makueni district and 53 in the Kitui district. Within those villages, we surveyed 705 households. Most [676 (96%)] of these households were randomly selected, and the remaining 29 (4%) were systematically selected households with an aflatoxicosis case-patient (cases who had been hospitalized) who was identified by hospital-based clinical disease surveillance in 2005 (Table 1). Eleven of the 705 households had maize from two different sources; therefore, we collected a total of 716 maize samples. We collected most (42%) of the 716 samples in 2005, followed by 35% in 2007, and 23% in 2006.

**Table 1:** Description of study sample by year.

Villages *n* (%)	Households *n* (%)	Maize samples *n* (%)
			Year
2005		36 (36)		289 (41)*a*		298 (42)
2006		38 (38)		160 (23)		165 (23)
2007		25 (25)		256 (36)		253 (35)
Total		99 (100)		705 (100)		716 (100)
**a**In 2005, the total number of households includes 260 households randomly selected and 29 households of case–patients identified by hospital reporting.						

*Maize production.* In 2005, most participants (96%) reported that their maize crop production was less than in previous years, and only 83 (28%) of the 298 maize samples collected were homegrown (grown on the household farm), whereas 215 (72%) were either purchased or obtained from the Kenyan government. In contrast, 97 (59%) of the maize samples collected in 2006 were homegrown. On average, households reported harvesting 328 kg (approximately four 90-kg bags) of maize in 2006. Maize production increased substantially in 2007 compared with the previous 2 years. All of the 256 samples collected in 2007 were homegrown, and none of the households reported purchasing maize or requesting relief maize. On average, households reported harvesting 1,169 kg (13 bags) of maize, or triple the harvest of 2006.

*Aflatoxin contamination.* For all 3 years combined (2005, 2006, and 2007), maize aflatoxin levels ranged from nondetectable to 48,000 ppb. The GM aflatoxin level for all 3 years combined was 9.10 ppb, and more than a third [248 (35%)] of the 716 maize samples had aflatoxin levels above the 20-ppb regulatory limit (Table 2). However, aflatoxin levels varied considerably by year, with GM levels decreasing dramatically in 2007 (GM = 1.95) compared with 2005 (GM = 12.92) and 2006 (GM = 26.03; *p*-value < 0.0001) (Table 2).

**Table 2:** Summary of the extent of aflatoxin contamination among all households, 2005–2007.

2005	2006	2007	All years combined
No. of samples tested		298		165		253		716
Overall GM (range)*a*		12.92 (0.11–48,000)		26.03 (0.30–24,400)		1.95 (< LOD–2,500)		9.10 (< LOD–48,000)
Households with aflatoxin								
< 20 ppb (%)		59		48		84		65
20–200 ppb (%)		23		28		13		21
201–1,000 ppb (%)		11		15		3		9
> 1,000 ppb (%)		7		8		0.4		5
**a**All estimates are adjusted for division and village using mixed linear models.								

We collected the maize sample with the highest aflatoxin level (48,000 ppb—2,400 times the regulatory limit for human consumption) in 2005, and the sample with the second highest level (24,400 ppb) in 2006. In the non-outbreak year (2007), contamination was much lower than in the previous 2 years; the two samples with the highest contamination had aflatoxin levels of 2,500 ppb and 470 ppb. In 2005 and 2006, 41% and 51% of samples, respectively, had aflatoxin levels above the 20-ppb regulatory limit compared with 16% of samples in 2007 (Table 2). In addition, 21 samples (7%), 14 samples (8%), and 1 sample (0.4%) had aflatoxin levels > 1,000 ppb in 2005, 2006, and 2007, respectively (Table 2).

*Source of maize and aflatoxin contamination.* We assessed and compared the extent of aflatoxin contamination in the three sources of maize in the region (homegrown, purchased, and relief) in 2005 and 2006 when all three sources of maize were collected from households and tested. In 2007, production was high, and all households had only homegrown maize. Most of the homegrown maize samples collected in 2005 and 2006 had aflatoxin levels above the 20-ppb regulatory limit (64% and 60%, respectively) compared with 41% and 40% of purchased maize samples (Table 3). Of the relief maize tested, three of 53 (6%) relief samples in 2005 and one of three (33%) in 2006 exceeded the 20-ppb limit. We found the highest levels of aflatoxin contamination in 2005 and 2006 (48,000 ppb and 24,400 ppb, respectively) in homegrown maize samples. Relief maize had considerably lower aflatoxin levels (see Table 3). In 2005, the aflatoxin level of homegrown maize (GM = 62.61) was significantly higher than the level of purchased maize (GM = 10.05) or of relief maize (GM = 1.99) (*p*-value < 0.0001). We also found similar, statistically significant differences in levels among the different sources in 2006 (*p*-value < 0.0001) (Table 4).

**Table 3:** Maize aflatoxin levels by source, 2005–2007.

Maize source	Samples with < 20 ppb [*n* (%)]	Samples with > 20 ppb [*n* (%)]	GM (range) of aflatoxin levels (ppb)*a*
2005						
Homegrown		30 (36)		53 (64)		62.61 (0.43–48,000)
Purchased		95 (59)		67 (41)		10.05 (0.11–13,800)
Relief		50 (94)		3 (6)		1.99 (0.37–720)
2006						
Homegrown		39 (40)		58 (60)		46.47 (0.49–24,400)
Purchased		39 (60)		26 (40)		14.57 (0.30–2,800)
Relief		2 (67)		1 (33)		6.21 (2.1–22)
2007						
Homegrown		213 (84)		40 (16)		1.95 (< LOD–2,500)
Purchased		–		–		–
Relief		–		–		–
All years combined						
Homegrown		282 (65)		151 (35)		17.96 (< LOD–48,000)*b*
Purchased		134 (59)		93 (41)		3.64 (0.11 – 13,800)*b*
Relief		52 (93)		4 (7)		0.73 (0.37 – 720)*b*
**a**All estimates are adjusted for division and village. **b**Estimates are adjusted for year.						

**Table 4:** Geometric mean aflatoxin levels in homegrown maize samples according to responses to aflatoxin awareness questions, 2005–2007.

Have you received information about how to dry and store your maize?			Do you think that eating moldy maize can cause muuku?			Did you hear about people getting muuku in this area from eating moldy maize?		
Year	*n* (%)	GM (ppb)	*n* (%)	GM (ppb)	*n* (%)	GM (ppb)
2005												
Yes		31 (38)		51.42		37 (62)		60.88		43 (54)		53.54
No		51 (62)		71.72		23 (38)		58.26		37 (46)		90.20
2006												
Yes		35 (36)		93.73		44 (63)		89.68		50 (54)		88.74
No		61 (64)		33.56		26 (37)		64.13		43 (46)		31.37
2007												
Yes		190 (74)		1.96		171 (82)		2.02		123 (49)		1.56
No		66 (26)		2.12		37 (18)		2.71		129 (51)		2.48
All years combined												
Yes		256 (59)		15.60		252 (75)		15.37		216 (51)		14.76
No		178 (41)		16.79		86 (25)		22.79		209 (49)		18.89


When comparing aflatoxin levels in homegrown maize between years, the GM homegrown maize aflatoxin levels were significantly higher in 2005 (GM = 17.96) and 2006 (GM = 3.64) compared with 2007 (GM = 0.73) (*p*-value < 0.0001). We found no significant difference in homegrown maize aflatoxin levels in 2005 compared with 2006.

*Aflatoxin contamination and jaundice.* We asked questions about the health of family members residing in the household, specifically, if any household members had symptoms of jaundice. Of the 696 randomly selected households in all 3 years combined (not including the aflatoxicosis case households), 13 (2%) reported that one or more family members residing in the household had symptoms of jaundice at the time the survey was conducted. The GM aflatoxin levels in maize being consumed by households reporting jaundice ranged from 4.02 to 18.57 ppb. The maximum aflatoxin levels found among households with jaundice was 820 ppb. No jaundice was reported among households consuming maize with aflatoxin levels > 1,000 ppb, including those consuming the highest concentrations (48,000 ppb, 24,000 ppb).

*Aflatoxin awareness.* When assessing aflatoxin awareness, we limited our analysis to the 436 respondents who had homegrown maize at the time of sampling to determine a potential association between level of awareness and aflatoxin concentrations in their homegrown maize. For all 3 years combined, 59% of our study participants with homegrown maize reported receiving information about drying and storing maize. There was a statistically significant increase in the percentage of people who reported receiving information in 2007 [190/256 (74%)] compared with 2005 [31/82 (38%)] and 2006 [35/96 (36%)] (*p*-value < 0.0001) (Table 4). However, we found no significant difference in GM aflatoxin levels between households that reported receiving information and those that did not (overall or by year). Although the difference was not statistically significant, in 2006 the GM aflatoxin levels were considerably lower in households that reported receiving information (Table 4).

We asked respondents if they thought eating moldy maize could cause muuku (jaundice); 252 (75%) respondents in all 3 years combined responded “yes” to this question. We found a statistically significant difference in percentage of those who reported thinking that jaundice may be caused by eating moldy maize when comparing 2005 and 2006 (62% and 63%, respectively) with 2007 (82%) (Table 4) (*p*-value < 0.0001). However, responses were not associated with statistically significant differences in GM aflatoxin levels (Table 4).

Finally, we asked respondents if they had heard about people getting muuku from eating moldy maize. In all 3 years combined, 216 (51%) of respondents answered yes to this question (Table 4). We found no statistically significant difference by year in proportions of those who responded yes to this question and no statistically significant differences in GM maize aflatoxin levels for those who responded yes compared with those who responded no.

## Discussion

Fungal contamination of home-stored subsistence crops such as maize is a global public health problem. However, there is little information about the concentrations of fungal toxins on foods actually eaten by families engaging in subsistence farming. A study conducted in Tanzania showed concentrations of aflatoxin on homegrown maize as high as 158 ppb (Kimanya et al. 2008). These levels were a significant concern; however, in the current study even higher concentrations were found in food stores in Kenya. This study suggests that consuming homegrown maize is a major source of aflatoxin exposure in the aflatoxicosis-prone southern region of Eastern Province of Kenya.

To minimize the potential human exposure to aflatoxins, the content of aflatoxin in food is regulated in most parts of the world (Shephard 2008; Van Egmond et al. 2007), including Kenya. Regulatory limits vary among countries, and none of the regulatory limits (including those in Kenya) are based on human health data. At the time of analysis, the Kenya regulatory limit for aflatoxin in food for human consumption was 20 ppb (Kenya Bureau of Standards 1988), which is also the FDA regulatory limit used in the United States (FDA 2000). However, Kenya has since reduced their regulatory limit to 10 ppb. Given that the consumption of maize in Kenya is high, making up 36% of the daily caloric intake of Kenyans (Gitu 2004), a lower limit for aflatoxin contamination in grains should be beneficial. However, the results of this study suggest that achieving the new 10-ppb limit will be a significant challenge for public health officials in Kenya.

Homegrown food consumed by subsistence farmers is not monitored for aflatoxin or other contaminants through traditional regulatory authorities. Researchers (Kimanya et al. 2008; Shephard 2003; Wild and Gong 2010; Williams et al. 2004) agree that people living in rural settings where locally grown crops are consumed should have enforced regulatory standards and other protective measures. Such measures could include providing agricultural extension officers to monitor food at the subsistence farm level, which may reduce the risk of exposure among these farmers (Redwooda Y, Lewisa L, Njapaub H, Breimanc R, Mwihiad J, Daniela J, et al., unpublished data), and could be coupled with interventions to help farmers properly store and dry their maize to reduce the risk of aflatoxin contamination.

Although homegrown maize had the highest levels of contamination and most samples in 2005 and 2006 exceeded the regulatory limit for aflatoxin, many purchased maize samples also had aflatoxin levels above the regulatory limit, some as high as 13,800 ppb in 2005. A previous study by Lewis et al. (2005) describing sources of maize found in local markets showed that homegrown maize may not remain within the household of a farmer after harvest. Farmers tend to sell maize to market vendors and purchase it back as needed. Therefore, the local trade and distribution of contaminated homegrown maize may facilitate and sustain the cycle of aflatoxin exposure for both farmers and the local community. The amount of exported maize or of aflatoxin-contaminated grain from this region and its effect on wider markets is unknown.

During the outbreak years, homegrown maize aflatoxin contamination was extremely high, whereas maize production was low or nonexistent. In 2007, when maize production dramatically increased, homegrown maize aflatoxin levels were low. The nature of crops under drought conditions may be the reason for this association. During periods of drought, crops are stressed and become more prone to aflatoxin contamination (Wilson and Payne 1994). In addition, food scarcity may promote improper farming practices such as harvesting maize while wet to ensure it would not be stolen (Azziz-Baumgartner et al. 2005), thus increasing the opportunity for fungal growth and aflatoxin contamination. Efforts to address food security during periods of drought should be linked to heightened food safety efforts to prevent consuming aflatoxin-contaminated food.

Although many maize samples had extremely high levels of aflatoxins, < 2% of the study population consuming this maize had jaundice at the time the questionnaire was administered. In fact, jaundice was not documented in households where maize levels were the highest (48,000 ppb and 24,400 ppb), highlighting that factors associated with developing aflatoxicosis after aflatoxin exposure are not fully characterized. Our findings suggest that chronic exposure to aflatoxin may be substantial and widespread in this region; the risk posed to public health is potentially great but not defined in this setting. The International Agency Research on Cancer (IARC) has classified aflatoxin as an established human carcinogen (IARC 1987), especially with co-existing chronic hepatitis B infection (IARC 1993; Wild and Montesano 2009; Williams et al. 2004). In the Kenyan population, in particular, it is estimated that approximately 70% of Kenyans have a positive hepatitis B virus serology by adulthood (Mphahlele et al. 2002). In addition, recent studies have shown decreased immune function among populations exposed to aflatoxin (Jiang et al. 2008). Although outbreaks of acute aflatoxicosis with associated high case-fatality rates command the attention and resources of governments and foreign aid agencies, assessing the effect of chronic exposure and implementing effective interventions to minimize chronic aflatoxin exposure is needed.

The Kenyan government expends substantial resources to educate farmers about the sources of aflatoxin contamination and proper drying and storing practices. Other studies have shown the benefits of education and awareness (Hell et al. 2000; James et al. 2007). Our study shows a significant increase of aflatoxin awareness over time. However, we found no significant association between maize aflatoxin levels and awareness, suggesting that the information provided may not translate to actions to change current maize harvest and storage practices. During drought periods, contamination may be more ubiquitous and beyond the control of individual farmers. Awareness of aflatoxin prevention methods without the accompanying agricultural infrastructure to effectively dry and store maize may not translate to behavioral changes that would prevent or mitigate the extent of contamination.

Our findings show that 35% of maize sampled in 2005, 2006, and 2007 exceeded the 20-ppb Kenyan regulatory limit with levels as high as 48,000 ppb. Previous studies suggest that the levels of aflatoxins found in homegrown maize in this region of Kenya are more than 60 times greater than the maximum maize aflatoxin levels found in other regions of the world. In a recent survey of published literature, we found a study by Williams et al. (2004) about aflatoxin contamination of market samples of foods from more than 20 countries. It showed that the maximum aflatoxin levels found in maize or corn products was 770 ppb in Nigeria, followed by 465 ppb in corn from Mexico (Williams et al. 2004). In additional studies done in neighboring Tanzania, aflatoxin levels in home-stored maize reached up to 158 ppb (Kimanya et al. 2008). Levels in other African regions measured in market stores reached 120 ppb in Benin, 490 ppb in Ghana, and 110 ppb in Togo (Hell et al. 2000; James et al. 2007). This demonstrates that the region of Eastern Kenya has a unique challenge with aflatoxins that deserves further exploration (Probst et al. 2007). Additionally, prevention efforts such as improving infrastructure for drying and storing and systematic screening and food monitoring may be necessary to minimize exposure to contaminated maize at the subsistence farm level.

*Limitations.* Our findings should be interpreted in light of some limitations. Our analysis included data collected from three individual public health responses. Sampling methodology varied by year. To address this, we conducted separate analyses by year, and when combining data from all 3 years we adjusted for year. In addition, the health information collected on the presence of jaundice was self-reported.

## Conclusions

Eating highly contaminated maize has resulted in outbreaks of acute aflatoxin poisoning for > 20 years in a localized area in Kenya (Azziz-Baumgartner et al. 2005; Ngindu et al. 1982). This study is the first to explore the potential sources of aflatoxin exposure by comparing levels in the various sources of maize available to this high risk population. These data show that homegrown maize had significantly higher aflatoxin levels than other sources (purchased or relief); however, purchased samples also had very high levels. Future prevention efforts to reduce aflatoxin exposure should target this high-risk population.

## Correction

In Table 4 of the manuscript originally published online, the values for *n* (%) were transposed for “All years combined.” The table has been corrected here.
